# Evolutionary mismatch between stomach capacity and modern diets: Implications for obesity and metabolic disease

**DOI:** 10.1093/emph/eoag020

**Published:** 2026-09-10

**Authors:** Mustafa Allouch, Philippe Attieh, Rawan Abdallah, Khaled El Jamal, Faten Mohtar, Bachir Zreika, Hani Kanso, Hilda E Ghadieh

**Affiliations:** Department of General Surgery, Faculty of Medicine and Medical Sciences, University of Balamand, Al-Koura, Tripoli, Lebanon; Chairman of Surgery, Hospital Nini, El Maarad Street, Tripoli, Lebanon; Department of General Surgery, Faculty of Medicine and Medical Sciences, University of Balamand, Al-Koura, Tripoli, Lebanon; Department of General Surgery, Faculty of Medicine and Medical Sciences, University of Balamand, Al-Koura, Tripoli, Lebanon; Department of General Surgery, Faculty of Medicine and Medical Sciences, University of Balamand, Al-Koura, Tripoli, Lebanon; Department of General Surgery, Faculty of Medicine and Medical Sciences, University of Balamand, Al-Koura, Tripoli, Lebanon; Department of General Surgery, Faculty of Medical Sciences, Lebanese University, Beirut, Lebanon; Department of General Surgery, Faculty of Medicine and Medical Sciences, University of Balamand, Al-Koura, Tripoli, Lebanon; Department of Biomedical Sciences, Faculty of Medicine and Medical Sciences, University of Balamand, Al-Koura, Tripoli, Lebanon

**Keywords:** evolutionary mismatch, stomach capacity, gastrointestinal anatomy, obesity, metabolic syndrome, bariatric surgery, evolutionary medicine

## Abstract

Human gastrointestinal anatomy evolved under conditions of food scarcity, low–caloric-density diets, and high physical activity. In this context, a relatively gastric-accommodation stomach capacity conferred a survival advantage by allowing consumption of large volumes of fibrous, low-energy foods, buffering seasonal shortages, and maximizing intake during rare periods of abundance. Modern food environments, characterized by constant access to energy-dense foods and sedentary lifestyles, have reversed these selective pressures, creating an evolutionary mismatch between ancestral digestive physiology and contemporary diets. This narrative review explores how a digestive architecture shaped by scarcity may become maladaptive in modern high-calorie conditions and examines stomach capacity as an under-recognized contributor to obesity and metabolic disease. Evidence was synthesized from evolutionary biology, anthropology, gastroenterology, and metabolic medicine using literature identified in PubMed, Scopus, and Web of Science (1990–2024). Evolutionary and anthropological evidence suggests that increased gastric volume was advantageous in both pre-agrarian and agrarian societies. In contrast, in modern environments this trait may promote excessive energy intake, delayed satiety, and metabolic dysfunction, particularly in the presence of ultra-processed foods. Clinical observations from bariatric surgery support this framework, as surgical reduction of gastric volume induces rapid metabolic improvements, often preceding significant weight loss. Gastric accommodation capacity, once adaptive, may now contribute to metabolic disease in obesogenic environments, reframing obesity as a predictable consequence of evolutionary mismatch.

## INTRODUCTION

Obesity and metabolic disease represent major and escalating global health challenges. Since 1975, obesity prevalence has nearly tripled, with >650 million adults now affected worldwide (World Health Organization, 2023). While obesity arises from complex interactions among biological, behavioral, environmental, socioeconomic, and developmental determinants, the evolutionary mismatch framework has gained prominence as a unifying explanatory model [1, 2]. Evolutionary mismatch refers to the discordance between ancient physiological adaptations and contemporary ecological conditions, particularly those marked by continuous caloric abundance, ultra-processed foods (UPFs), reduced physical activity, and profound shifts in food environments.

One under-recognized dimension of evolutionary mismatch is stomach capacity. Across clinical and bariatric research, a consistent observation is that gastric volume influences feeding behavior and satiety. Imaging-based studies using techniques such as single photon emission computed tomography (SPECT) demonstrate that gastric volume and accommodation can be reliably quantified in vivo and vary substantially between individuals [3]. Over millions of years, early hominin diets were ecologically variable but often included fibrous plant foods such as underground storage organs, leaves, stems, fruits, and nuts, supplemented intermittently with animal resources [4–6]. Under these conditions, relatively large gastric capacity facilitated bulk ingestion, prolonged retention, mechanical processing, and microbial fermentation, improving caloric yield and buffering survival during periods of scarcity.

Agricultural societies preserved selective pressures for high gastric throughput. Grain-based staple diets demanded large-volume ingestion to meet caloric needs, especially during periods of strenuous physical labor and seasonal famine cycles [7, 8]. In contrast, modern dietary environments invert ancestral constraints: food is abundant, highly energy-dense, low in fiber, and rapidly ingested, while daily physical activity has declined dramatically [9, 10]. These changes render previously adaptive traits maladaptive. Large gastric capacity now facilitates rapid meal ingestion, delayed satiety signaling, and chronic caloric surplus, especially when combined with UPFs that bypass key mechanosensory pathways and reduce gastric distension.

Bariatric surgery provides clinical lens for understanding this mismatch. Procedures such as sleeve gastrectomy markedly reduce gastric volume and modify nutrient flow and gut–brain signaling. Metabolic improvements, including type 2 diabetes remission and enhanced incretin secretion—often arise before significant weight loss, highlighting the central role of gastric volume, stretch signaling, and nutrient–gut interactions in metabolic regulation [11, 12]. These findings suggest that gastric morphology and accommodation are potent modulators of appetite, energy intake, glucose homeostasis, and long-term metabolic risk.

The present narrative review synthesizes evidence from evolutionary biology, anthropology, gastroenterology, nutritional science, and metabolic medicine to evaluate the hypothesis that stomach capacity represents an under-appreciated evolutionary determinant of modern metabolic disease. The analysis is conceptual and hypothesis-generating. It does not advocate specific clinical interventions, nor does it interpret bariatric surgery as a restoration of ancestral physiology. Rather, it uses surgical outcomes and comparative anatomy as probes to better understand how ancient digestive architecture interacts with contemporary food ecologies.

The present review integrates multiple lines of evidence, including comparative and evolutionary biology, observational data from traditional and modern human populations, mechanistic studies of gastric physiology and gut–brain signaling, and clinical and interventional evidence from bariatric surgery and metabolic research.

## METHODS

Narrative review methodology was adopted due to the interdisciplinary nature of the topic, which spans evolutionary biology, anthropology, gastroenterology, and metabolic medicine. A comprehensive search was conducted across PubMed, Scopus, and Web of Science. Eligible studies included peer-reviewed publications from 1990 to 2024 focusing on human physiology, anthropological evidence, clinical research on gastric volume, metabolic health, and reviews related to evolutionary diet and metabolism. Exclusion criteria comprised non-human animal studies unrelated to human evolution, non–peer-reviewed sources, and articles lacking clear methodological descriptions. All included studies were synthesized to identify cross-disciplinary patterns and insights relevant to the subject. A narrative search differs from a systematic review in that it does not aim to identify all eligible studies through exhaustive and reproducible search strategies, nor does it include formal risk-of-bias assessment or quantitative synthesis. Instead, it allows selective inclusion of studies from diverse disciplines to support conceptual integration and hypothesis development, which is appropriate for interdisciplinary and theory-generating topics such as evolutionary mismatch.

### Literature search and selection

A structured narrative search was conducted in PubMed, Scopus, and Web of Science to identify relevant literature across evolutionary biology, anthropology, gastroenterology, nutrition science, and metabolic medicine. Example PubMed search syntax included combinations of the following terms:

(“stomach capacity” OR “gastric volume” OR “gastric accommodation” OR “gastric distension”) AND (“satiety” OR “food intake” OR “energy intake” OR “obesity” OR “metabolic”) AND (“evolution*” OR “evolutionary mismatch” OR “hunter-gatherer” OR “agriculture” OR “diet evolution” OR “bariatric surgery”).

The primary search window was 1990–2024. This period was selected because it corresponds to [1] the emergence and expansion of modern metabolic–bariatric surgery [2], the availability of contemporary imaging and physiological methods for measuring gastric volume and accommodation, and [3] the development of current concepts regarding UPFs and modern dietary environments. Earlier foundational works in evolutionary anthropology and human biology were included when directly relevant.

The search yielded ~450–600 records across databases. After removal of duplicates, titles and abstracts were screened for relevance to human gastric physiology, feeding behavior, evolutionary diet, or metabolic outcomes. Approximately 120–150 articles were reviewed in full text, and ~35–45 key sources were selected for inclusion based on relevance, methodological clarity, and conceptual contribution.

Studies were included if they addressed:

- human gastric size, accommodation, or distension
- satiety mechanisms, gut–brain signaling, or energy intake
- anthropological or evolutionary evidence related to diet and digestive adaptation
- metabolic outcomes associated with gastric restriction or bariatric surgery

Mechanistic animal studies were included when directly relevant to physiological mechanisms (e.g. vagal afferent signaling, stretch sensitivity, or gut–brain regulation of satiety), but studies unrelated to human physiology or evolutionary interpretation were excluded.

This approach was intended to identify cross-disciplinary patterns relevant to the proposed evolutionary mismatch framework rather than to provide an exhaustive systematic review.

## PRE-AGRARIAN EVIDENCE: SCARCITY, FIBROUS DIETS, AND LARGE GASTRIC CAPACITY

### Ecological scarcity and dietary constraints

Because soft tissues do not fossilize, direct measurement of gastric size in early hominins is unavailable, but comparative primate anatomy provides an informative proxy. Among great apes, gut proportion and stomach volume strongly track dietary niche: frugivores such as chimpanzees have large, sacculated stomachs, while folivores such as gorillas possess even more expanded gastrointestinal tracts optimized for microbial fermentation and bulk vegetation processing [4, 13]. Early hominin diets likely varied across time and ecological context but included underground storage organs, leaves, stems, fruits, nuts, and intermittent animal foods [5, 6]. These resources often required substantial mechanical processing and variable intake volume, consistent with a digestive system adapted to dietary intermittency and ecological variability.

The evolutionary interpretation presented here is inferential and based on ecological plausibility rather than direct evidence of selection on gastric size, which cannot be reconstructed from the fossil record.

### Fasting–gorging cycles and opportunistic feeding

Scavenging and opportunistic hunting produced episodic access to calorie-dense resources—marrow, organ meats, fat, honey [13]. Greater gastric accommodation would have allowed rapid ingestion of large quantities when high-value resources were available, before losing access due to predators, competition, or carcass decay. In these contexts, time was a critical constraint since carcasses were often contested by carnivores, while honey or fat deposits were seasonally unpredictable and quickly depleted. Individuals who could consume more calories in a short window gained a selective advantage, not only because they could store immediate energy, but also because the absorbed nutrients supported thermoregulation, brain metabolism, and reproductive success. Rapid ingestion reduced exposure to predation during feeding and maximized the caloric return from rare, high-value resources that might not be encountered again for days or weeks.

### Ancestral retention rather than anatomical enlargement

Modern humans do not possess an unusually large stomach relative to body size compared with other primates. Instead, evolutionary evidence suggests that early *Homo* experienced a reduction in total gut volume associated with increased dietary quality, meat consumption, and the energetic effects of cooking, consistent with the expensive-tissue hypothesis [14, 15]. However, humans retain substantial gastric accommodation capacity, reflecting a flexible physiological system adapted to intermittent intake and variability in food volume. The evolutionary mismatch proposed here therefore reflects ecological change rather than anatomical enlargement: a digestive system calibrated for variable-volume, lower–energy-density foods now operating in environments dominated by continuously available, energy-dense diets.

### Strategic importance of stomach capacity

Under harsh ecological variability, a large stomach provided buffering capacity. Greater gastric accommodation may have provided buffering capacity by allowing larger meal volumes during periods of unpredictable food availability, as hominins relied on irregular access to high-quality foods and needed to ingest large quantities when available [4, 5]. It gave metabolic stability across seasons, since larger gastric volume facilitated the consumption and slow processing of bulky, fibrous fallback foods during periods of scarcity [6, 13]. Moreover, increased survival during lean periods by enabling prolonged nutrient extraction and supporting greater tolerance of low-quality plant matter during ecological downturns [14]. This digestive architecture complemented broader evolutionary strategies of early *Homo*, including technological innovation, increased mobility, and dietary flexibility, which together enhanced resilience in unstable, resource-limited environments [9, 15].

The framework presented here does not imply direct selection on stomach size per se, but rather that variation in gastric accommodation capacity would have been advantageous under ecological conditions characterized by intermittent access to bulky or unpredictable food resources.

## EVIDENCE FROM AGRARIAN SOCIETIES

Agriculture introduced predictable harvests yet unpredictable shortages, monotony of cereal-based diets, and intense physical labor, all of which maintained selective pressure for high gastric throughput**.** It also transformed social and cultural habits exemplified in the shift to sedentism, the storage of surplus grains, and the emergence of stratified labor systems reorganized daily routines and food practices [8]. Communal eating schedules, seasonal feasting, and long intervals of labor between meals became embedded features of agrarian life. These patterns reinforced large-volume consumption and created enduring patterns of work, diet, and social organization that shaped habitual metabolic regulation calibrated to periodic feeding and seasonal scarcity, which persisted for millennia [8].

### Dietary monotony and bulk consumption

Early agricultural diets were dominated by cereal staples, wheat, rice, and maize, often constituting up to 80% of caloric intake [8]. These foods were bulky and low in nutrient density. A larger stomach improved the ability to ingest sufficient calories despite low protein/fat content, because grain-based diets required high-volume consumption to compensate for limited amino acids, fats, and micronutrients. It also allowed processing coarse, fibrous grains**,** which demanded prolonged gastric mixing, hydration, and mechanical digestion due to their toughness and high phytate content [7]. Moreover, it allowed maintaining energy levels during labor-intensive agricultural tasks**,** as the ability to consume large meals during short rest intervals supported the heavy physical workloads characteristic of early farming communities [16]. These conditions likely favored tolerance for high-volume intake and efficient processing of bulky cereal-based diets, although direct evidence of selection on gastric capacity during the agrarian period is limited [17].

### Seasonal famine cycles (“hungry seasons”)

Reliance on annual harvests created predictable cycles. Post-harvest abundance-feasting, and pre-harvest scarcity-famine.

Individuals with larger stomachs could ingest large quantities post-harvest to build fat reserves, taking advantage of brief periods of caloric abundance to accumulate adipose stores that would sustain them through the coming “hungry season” [8, 17, 18]. They could, therefore, have a better survival in prolonged seasonal deficits**,** since increased gastric volume allowed more efficient processing of bulky, low-quality staples and extended nutrient release during periods when food availability declined [7]. A larger stomach can help to tolerate fallback foods (wild grasses, roots, weeds**)**, which were fibrous, coarse, and often difficult to digest, requiring greater gut capacity to extract sufficient energy [5]. These dynamics reinforced metabolic thriftiness and digestive capacity, as populations regularly cycling between abundance and scarcity experienced strong selective pressures favoring traits that supported efficient energy storage, high-volume ingestion, and tolerance of low-nutrient diets [1].

### High physical labor demands

Farming requires continuous high-intensity labor. Therefore, a larger stomach conferred a capacity for large meals during limited breaks**,** as agricultural work often involved long hours in the fields with few opportunities to eat, favoring individuals who could consume substantial calories quickly and sustain prolonged exertion [16]. It also allowed improved carbohydrate extraction from grains, since larger gastric volume enhanced the hydration, breakdown, and enzymatic processing of coarse cereals, which were the dominant energy source in early farming societies [7]. In addition, it ensured better metabolic buffering during seasonal workloads**,** helping individuals maintain stable energy output during periods of peak agricultural demand such as planting and harvest seasons [8]. This situation produced a labor–nutrition–physiology feedback loop, in which strenuous agricultural activity increased caloric needs, high-volume meals met those demands, and individuals with greater digestive throughput were better able to withstand the metabolic strain. Over generations, these conditions reinforced traits that supported high caloric intake, efficient carbohydrate processing, and endurance under sustained physical labor [14].

## EVIDENCE FROM MODERN SOCIETIES

Modern environments invert ancestral conditions, leading to mismatch**.** Instead of intermittent scarcity, humans now encounter continuous food availability; instead of fibrous, low-calorie foods, diets are dominated by ultra-processed, energy-dense products; and instead of high physical activity, most daily life is sedentary. These reversals disrupt the physiological cues and digestive mechanisms that evolved under scarcity, creating systematic conflicts between ancient adaptations and modern ecological realities. Early quasi-experimental evidence also supports the role of lifestyle factors in shaping metabolic risk, demonstrating that changes in diet and physical activity can rapidly influence metabolic outcomes even in relatively short time frames [19].

### Food abundance, high caloric density, and satiety disruption

UPFs are soft, low in fiber, rapidly ingested, and usually extremely calorie-dense. These characteristics reduce chewing and gastric distension, delaying satiety and accelerate caloric intake. Because they require minimal oral processing and create little gastric stretch, UPFs fail to adequately activate the mechanical and hormonal satiety pathways that evolved to regulate intake under high-fiber, low-calorie ancestral diets**.** Galdino-Silva *et al.* (2024) demonstrated that UPF meals lead to faster eating rates, fewer chews per bite, and a smaller reduction in the physiological “capacity to eat,” indicating weaker satiety signaling during the meal [20].

Mechanosensory pathways further compound this problem. Kentish *et al.* (2015) showed that obesity is associated with blunted activation of gastric vagal afferents, stretch-sensitive neurons that normally signal fullness, reducing the body’s ability to detect gastric distension [21]. Similarly, Geliebter (2013) reported that individuals with higher body mass index (BMI) tolerate substantially greater gastric volume before exhibiting satiety-related brain responses [22]. Together, these findings illustrate how UPFs bypass or weaken the evolutionarily conserved satiety mechanisms that depend on chewing, gastric stretch, and slow nutrient processing, thereby promoting rapid overconsumption and positive energy balance.

### Sedentary life

Modern sedentary behavior reduces energy expenditure far below ancestral norms [23]. Pontzer’s constrained total energy expenditure model demonstrates that the body maintains metabolic expenditure within a narrow range, limiting the impact of exercise on total caloric burn [9]. In this framework, the human body adapts to low activity by downregulating other components of daily energy expenditure, such as immune activity, stress responses, and tissue maintenance, so that total caloric burn remains relatively constant regardless of moderate increases in physical activity. Thus, high intake + low expenditure = chronic positive energy balance, because even modest caloric surpluses accumulate when the body’s energy output is physiologically capped. Large stomach capacity facilitates excess intake in this environment, as greater gastric accommodation allows rapid consumption of calorie-dense foods without triggering early satiety, making it easier to surpass the limited metabolic ceiling imposed by the constrained energy model. Together, these factors create a metabolic landscape in which ancestral adaptations for high-volume intake collide with modern sedentary lifestyles, amplifying the risk of obesity and metabolic disease.

### Bariatric surgery: physiological insight into evolutionary mismatch

Bariatric procedures dramatically reduce gastric capacity and modify gut–brain hormonal signaling. Sleeve gastrectomy removes ~75%–80% of the stomach, including ghrelin-rich fundus tissue [24]. Outcomes include immediate reduction in meal size, because the remaining gastric sleeve has far lower distensibility and reaches satiety-inducing stretch thresholds quickly, and earlier activation of satiety mechanisms**,** as reduced gastric volume accelerates nutrient delivery to the small intestine, triggering rapid secretion of incretin hormones. This may allow durable long-term weight loss through sustained reductions in intake and changes in appetite regulation [25].

Critically, metabolic improvements occur rapidly, often within days to weeks, before major weight loss. This early effect highlights the central role of gut-derived hormonal and neural pathways, not merely fat mass reduction, in restoring metabolic control. Randomized trials post metabolic-bariatric surgeries show high rate of remission of type 2 diabetes**,** frequently occurring before meaningful weight change, along with improved insulin sensitivity, driven by altered nutrient flow and incretin responses, and enhanced GLP-1 and PYY secretion, potent satiety hormones that improve glucose homeostasis, all independent of significant fat loss [11, 12]. However, it is important to note that surgery is a perturbation, not an ancestral proxy.

## EVOLUTIONARY ADVANTAGE BECOMES LIABILITY

Human gastrointestinal morphology reflects millions of years of adaptation to scarcity, ecological variability, and high energy expenditure. Under ancestral environments, greater gastric accommodation would have facilitated the ingestion of bulky, fibrous foods, which required substantial gastric volume for hydration, fermentation, and prolonged mechanical breakdown given their low nutrient density [5]. Also, extraction of calories from nutritionally dilute plant materials, enabling early hominins to utilize fallback foods and survive periods of ecological stress [4]. It also allowed exploitation of rare caloric windfalls**,** as rapid ingestion of marrow, fat, or honey maximized caloric gain before competitors or scavengers arrived. This likely improved survival through recurrent cycles of famine**,** with large gastric capacity facilitating both large post-feast intake and prolonged nutrient release during scarcity [14].

Importantly, the evidence linking baseline gastric volume to obesity and metabolic risk in free-living human populations is largely indirect but convergent rather than definitive. Current data are drawn from comparative primate anatomy, cross-sectional human studies of gastric capacity and food intake, and metabolic outcomes after bariatric surgery, whereas longitudinal studies directly testing whether larger gastric capacity predicts future weight gain or metabolic disease remain scarce. It is worth noting that agrarian diets, though bulky and high in carbohydrates, were not ultra-processed; the current crisis arises when that gastric architecture meets soft, highly processed, low-fiber, hyper-palatable foods.

Comparative primate evidence should be interpreted as supporting an ancestral baseline rather than a modern anatomical match: early hominins likely possessed greater gastric and intestinal accommodation than is typical today, but human digestive morphology subsequently contracted with cooking and higher diet quality. The relevance to modern obesity lies in the evolutionary legacy of digestive tolerance for large-volume intake, not in assuming that the contemporary human stomach resembles the gorilla foregut. These traits enhanced survival and reproductive fitness in environments where food availability was unpredictable, and energy demands were high.

In modern environments, characterized by abundant, hyper-palatable, calorie-dense foods and sedentary lifestyles, the same traits have become maladaptive. A larger stomach facilitates greater meal volume, as individuals can easily ingest more calories before reaching fullness, delayed satiety due to reduced gastric stretch, especially when consuming soft or liquid UPFs that produce minimal distension, accelerated caloric intake when consuming UPFs**,** which require little chewing and allow rapid ingestion, bypassing key satiety cues [10]. This resulted in chronic positive energy balance, as caloric intake routinely exceeds the constrained total energy expenditure of sedentary lifestyles [9].

This promotes adiposity, insulin resistance, dyslipidemia, and non-alcoholic fatty liver disease [26]. The result is a classic evolutionary mismatch: traits shaped to secure survival under scarcity now predispose individuals to metabolic disease when confronted with continuous caloric abundance, low-fiber diets, and minimal physical activity [1, 2].

## MOTH-TO-THE-FLAME ANALOGY: EVOLUTIONARY LAG IN MODERN ENVIRONMENTS

The evolutionary predicament of human digestive physiology resembles the well-known trap of moth phototaxis. For millions of years, moths used the moon and stars, distant, stable light sources to navigate. Artificial lights disrupt this ancient cue, causing moths to spiral into flames. Their navigational program is not defective; it is simply operating in an environment for which it was never designed.

Human physiology behaves analogously. Mechanisms regulating hunger, reward, gastric stretch, and satiety evolved under conditions of scarcity, low-calorie foods, and intermittent access to high-value nutrients. These heuristics were highly adaptive when food was rare, fibrous, and difficult to obtain. In modern environments where food is abundant, hyper-palatable, energy-dense, and continuously accessible, the same physiological cues become liabilities. Large gastric capacity facilitates rapid intake and delayed satiety, while UPFs bypass oral processing and gastric distension, undermining mechanisms that evolved to limit intake under ancestral conditions.

In this sense, obesity does not represent a biological failure, but an evolutionary lag: physiology calibrated for scarcity is now operating in perpetual abundance. Just as the moth’s visual system cannot adjust its compass for artificial lights, the human digestive and metabolic system cannot easily recalibrate to modern caloric environments. Our ancestral appetite and gastric morphology respond as if we still inhabit a world of famine risk, high energy expenditure, and dietary scarcity. This is why traits such as large stomach capacity, adapted for scarcity, now heighten vulnerability to obesity, insulin resistance, type 2 diabetes, dyslipidemia, NAFLD, metabolic syndrome, and predisposition to cardiac and malignant diseases, among many.

## THRIFTY GENOTYPE AND THRIFTY PHENOTYPE INTERACTIONS

In this context, “metabolic thrift” refers to physiological mechanisms that reduce energy expenditure and promote efficient energy storage during periods of limited food availability.

Stomach capacity interacts synergistically with evolutionary metabolic thriftiness.

### Thrifty genotype (Neel, 1962)

Ancient humans have likely evolved rapid insulin responses, which facilitated swift clearance of glucose from the bloodstream following rare episodes of caloric abundance. Such as successful hunts or seasonal fruit availability, thereby maximizing conversion of nutrients into stored energy [18]. This allowed efficient fat storage, enabling individuals to deposit surplus calories into adipose tissue during times of plenty and rely on these reserves during predictable periods of famine or elevated physical activity [27]. During fasting, adaptive energy conservation occurs, characterized by reductions in basal metabolic rate and relative peripheral insulin resistance, which preserve glucose availability for vital organs [28].

In modern caloric abundance, these same traits become maladaptive. Continuous access to energy-dense foods produces chronic stimulation of insulin secretion, leading to hyperinsulinemia**,** as the rapid insulin response was once protective, now drives excessive glucose uptake and fat storage. Modern diet drives for adipose accumulation because efficient fat storage pathways are activated repeatedly and without the balancing effect of famine or high-energy expenditure. Eventually, this contributes to the emergence of metabolic syndrome, as physiological adaptations that conserve energy during fasting become maladaptive in environments where fasting is rare, promoting insulin resistance, dyslipidemia, and chronic inflammation. Thus, the thrifty genotype, originally selected to enhance survival under conditions of scarcity, now contributes to the rising global burden of obesity and type 2 diabetes when exposed to continuous caloric abundance, sedentary lifestyles, and UPFs. In this sense, Neel’s hypothesis provides a foundational evolutionary explanation for why modern metabolic disease emerges from biological traits that were once essential for human survival.

### Thrifty phenotype (Hales & Barker, 1992)

Early-life undernutrition programs individuals for metabolic efficiency, probably reduced β-cell mass, as inadequate fetal and early postnatal nutrition impairs pancreatic development, limiting insulin secretory capacity throughout life. This means heightened insulin resistance as an adaptive response in nutrient-poor environments that prioritizes delivery of glucose to vital organs, such as the brain and heart, at the expense of peripheral tissues. It also meant energy-conserving physiology**,** including lower basal metabolic rate, preferential fat storage, and hormonal adjustments that reduce energy expenditure and enhance survival during recurrent scarcity. These adaptations are advantageous in environments where food remains limited, as they help preserve critical metabolic functions and extend survival during famine.

However, when individuals programmed for scarcity are later exposed to caloric abundance, these same traits become harmful. Reduced β-cell reserve limits their ability to manage chronic high glucose loads, while baseline insulin resistance predisposes them to hyperglycemia, compensatory hyperinsulinemia, and rapid fat accumulation. Energy-conserving physiology amplifies this effect by channeling caloric surplus toward adipose tissue more efficiently than in individuals without early-life programming. Consequently, people who experienced fetal growth restriction, low birth weight, or early childhood malnutrition face substantially increased risk of obesity, type 2 diabetes, and metabolic syndrome in adulthood when exposed to modern nutrient-rich environments. This embodies the essence of the thrifty phenotype hypothesis as a physiology optimized for scarcity becomes a liability in abundance [29].

### Interaction with gastric capacity

When thrifty programming encounters a large stomach, meal size tends to increase, because individuals with energy-conserving physiology can tolerate greater gastric volume and reach fullness more slowly, especially when consuming modern calorie-dense foods [28]. **S**atiety is delayed, as both the enlarged stomach and developmental programming blunt mechanosensory and hormonal satiety cues, leading to prolonged eating episodes [1]. Then excess calories are preferentially stored as fat**,** since thrifty phenotypes display heightened insulin sensitivity in adipose tissue and reduced basal metabolic rate, channeling surplus energy efficiently into long-term storage. As a result, metabolic disease risk intensifies, as repeated cycles of high intake and efficient storage promote the rapid onset of obesity, insulin resistance, dyslipidemia, and impaired glucose tolerance [30]. In this interaction, the combination of large gastric capacity and thrifty metabolic programming creates a synergistic vulnerability to modern food environments. The stomach enables substantial caloric ingestion, while the thrifty phenotype ensures that even modest surpluses are stored aggressively. This convergence magnifies the mismatch between ancient adaptive traits and today’s continuous caloric abundance.

Thus, obesity emerges as the product of multiple evolutionary legacies. This includes digestive morphology shaped by intermittent scarcity, genetic selection for metabolic thrift, and developmental programming under early-life undernutrition. These once-advantageous traits now act in concert to increase susceptibility to metabolic disease when exposed to modern diets and sedentary lifestyles. It is also worth mentioning that gastric capacity may be one morphological “module” that amplifies thrifty genotype/phenotype effects, rather than an entirely separate axis. And this paper is proposing gastric capacity as a *neglected third component* in the thrifty narrative, alongside genetic and developmental programming.

## WHAT THIS PAPER IS NOT CLAIMING (CLARIFYING SCOPE)

To avoid misinterpretation, several boundaries of this analysis must be explicit.

1. Gastric capacity is not the sole cause of obesity.
2. Obesity arises from a complex interplay of dietary patterns, behavioral factors, genetic variance, endocrine regulation, developmental programming, socioeconomic context, and built environments [31]. Gastric morphology contributes to energy intake and satiety but represents only one component within a multifactorial system. Environmental drivers, especially UPFs, reduced physical activity, and social determinants of health, remain central forces shaping global obesity trends.
3. Not all individuals with obesity have large stomachs.
4. There is substantial heterogeneity in gastric accommodation, gastric emptying rates, vagal stretch receptor sensitivity, and gut–brain hormonal signaling [21, 22]. Some individuals with obesity exhibit normal or even reduced gastric capacity, while others show impaired mechanosensory satiety signaling independent of stomach size. This variability underscores that obesity cannot be reduced to anatomical differences alone. Nevertheless, clinical experience suggests that some individuals with obesity who consume large meal volumes may exhibit greater gastric accommodation; however, systematic evidence linking habitual meal size to baseline gastric capacity remains limited.
5. Bariatric surgery does not restore an “ancestral digestive system.”
6. Its metabolic benefits arise not from returning the gut to an ancient form, but from modern surgical alterations that dramatically modify anatomy, nutrient flow, and hormonal signaling. Procedures such as sleeve gastrectomy and Roux-en-Y gastric bypass produce enhanced GLP-1 and PYY secretion, reduced ghrelin production due to fundus removal, accelerated nutrient delivery to distal intestine, and changes in bile acid metabolism and gut microbiota composition [11, 12].
7. These effects reflect contemporary surgical physiology, not evolutionary reversion. The post-operative gastrointestinal tract is a novel configuration that did not exist in ancestral environments and cannot be interpreted as a restoration of prehistoric digestive traits.

## WHAT THE PAPER DOES ARGUE

### Gastric capacity is an under-recognized but critical contributor to the evolutionary mismatch framework.

The human stomach evolved to accommodate large volumes of low-calorie, fibrous foods and to exploit episodic access to calorie-dense windfalls [4, 5]. In modern environments, this same capacity enables rapid consumption of ultra-processed, energy-dense foods that fail to trigger early satiety signals, thereby exacerbating chronic caloric surplus. Despite its centrality to feeding behavior, gastric volume is seldom emphasized alongside well-known mismatch factors such as physical inactivity, ultra-processed diets, or obesogenic environments [1]. Recognizing gastric capacity as an evolutionary legacy provides an additional explanatory layer in understanding vulnerability to obesity.

Bariatric outcomes, especially improvements occurring before weight loss, demonstrate that gastric size and gut–brain interactions meaningfully regulate metabolic homeostasis.

Clinical trials consistently show that type 2 diabetes remission, improved insulin sensitivity, and enhanced incretin secretion occur within days to weeks after bariatric surgery, long before significant fat loss [11, 12]. These rapid metabolic changes highlight the crucial role of gastric anatomy and nutrient–gut interactions in modulating glucose homeostasis, appetite regulation, and hormonal signaling. Such evidence supports the argument that gastric volume, and its downstream neuroendocrine effects, is a potent regulator of metabolic physiology, not merely a passive reservoir for food.

### Viewing obesity through an evolutionary lens reframes it as a predictable outcome of ancient physiology meeting modern abundance

Humans possess metabolic traits exemplified in efficient fat storage, rapid insulin responses, preference for energy-dense foods that evolved to ensure survival under scarcity [2, 28]. When these traits encounter environments characterized by continual caloric availability, minimal physical activity, and UPFs engineered for palatability, obesity becomes an expected by-product rather than a failure of individual choice. Framing obesity as an evolutionary mismatch shift focusses from personal responsibility toward structural, biological, and ecological determinants, aligning with contemporary perspectives in evolutionary medicine [14].

## PUBLIC HEALTH IMPLICATIONS

Understanding stomach capacity as part of the evolutionary mismatch reframes obesity as a structural and ecological issue rather than an individual behavioral problem. This perspective emphasizes modifying food environments, developmental exposures, and satiety cues that interact with ancient physiological traits. Several implications follow:

1. Dietary Guidelines: Emphasize foods that fill gastric volume without excessive caloric density, particularly high-fiber, low-energy-density foods such as vegetables, legumes, and whole grains, which promote gastric distension, slow nutrient absorption, and enhance satiety [32]. Reduce liquid calories and hyper-palatable UPFs that bypass oral processing and gastric stretch, undermining satiety mechanisms and facilitating rapid caloric intake [10].
2. Portion Control Policies: Standardize serving sizes to counteract “portion distortion,” which exploits gastric accommodation capacity and normalizes excessive meal volumes [33]. Restrict supersized beverage and meal options that deliver large caloric loads in brief eating episodes. Apply evidence-based design interventions in smaller plates, reduced utensil size, and meal-structure nudges that have been shown to reduce intake without compromising satiety [34].
3. Environment-Level Interventions: Implement zoning restrictions on fast-food density in high-risk neighborhoods, reducing default access to high-calorie, low-satiety meals [35]. Provide incentives for supermarkets and fresh food vendors in underserved areas to increase access to nutrient-dense, high-volume foods. Promote industry reformulation targets that lower caloric density and increase fiber content, aligning modern food properties with evolved satiety pathways [36].
4. Early-Life Nutrition Programs: Strengthen maternal and childhood nutrition policies to reduce early-life undernutrition and prevent thrifty-phenotype programming, which increases metabolic disease risk in adulthood [1, 30]. Encourage breastfeeding, diverse whole-food introduction, and micronutrient sufficiency to support healthy metabolic and gut–brain developmental trajectories.
5. Bariatric Surgery Guidelines: Align appropriate indications for metabolic surgery, recognizing that surgical reduction of gastric volume and modification of gut–brain signaling provides powerful therapeutic benefit in modern obesogenic environments [12, 37]. Frame metabolic surgery as a targeted intervention addressing maladaptive gastric capacity, not as restoration of an ancestral state, since post-operative gut anatomy produces novel hormonal and metabolic dynamics [11]. It is important to note that in this paper we describe implications but do not recommend surgery as a population solution.

### Implications for “metabolic” indications

Our framework also bears on a practical question: whether operations that reduce gastric capacity should be understood as treating metabolic disease rather than obesity alone. Clinically, this shift is already reflected in the concept of metabolic surgery, where contemporary guidance recommends metabolic/bariatric surgery for BMI ≥35 kg/m^2^ regardless of comorbidity severity and supports consideration in selected patients with BMI 30–34.9 kg/m^2^ and metabolic disease (notably type 2 diabetes) when adequate metabolic control is not achieved with nonsurgical therapy. Importantly, the present paper does not propose new indications. Instead, it offers a mechanistic-evolutionary rationale for why modifying gastric capacity and nutrient–gut signaling can produce rapid metabolic effects, and why similar targets may increasingly be addressed via non-surgical modalities such as incretin-based pharmacotherapy. Decisions about surgery vs pharmacotherapy remain individualized and guideline-governed.

## LIMITATIONS AND FUTURE RESEARCH

This review acknowledges several limitations:

1. Soft tissue non-preservation: Gastric size cannot be directly measured in fossil hominins; inferences rely on comparative primatology and thoracic morphology.
2. Inter-individual heterogeneity: Modern variation in gastric capacity, satiety signaling, and metabolic phenotypes complicates generalization.
3. Complexity of obesity: Gastric morphology interacts with microbiome composition, endocrine regulation, early-life programming, epigenetics, and food environments.
4. Need for modern imaging studies: Future research should employ magnetic resonance imaging (MRI), computed tomography (CT) volumetry, and ultrasonography to quantify gastric volume variation across populations.
5. Need for cross-cultural longitudinal studies: Understanding how lifestyle, diet, and gastric capacity interact across societies will expand applicability.
6. Direct, longitudinal evidence linking baseline gastric volume to future obesity risk is limited; most data come from cross-sectional clinical studies and bariatric interventions.

Despite limitations, the integration of evolutionary biology, digestive physiology, and metabolic medicine provides a coherent framework for understanding modern disease patterns.

## TESTABLE PREDICTIONS AND RESEARCH DIRECTIONS

If gastric accommodation capacity contributes to evolutionary mismatch in modern environments, several empirically testable predictions follow.

### Prospective risk prediction

Individuals with greater baseline gastric accommodation or distensibility should exhibit higher long-term risk of weight gain and metabolic disease when exposed to energy-dense diets, independent of baseline BMI. Longitudinal cohort studies using MRI, barostat testing, or ultrasonographic volumetry could directly evaluate this prediction.

### Environment–physiology interaction

The relationship between gastric capacity and energy intake should be stronger in dietary environments characterized by high energy density and UPFs, and weaker in high-fiber, low–energy-density dietary contexts. Controlled feeding studies manipulating dietary energy density could test whether gastric accommodation predicts differential intake across food environments.

### Cross-population variation

Populations consuming traditional high-fiber diets should exhibit weaker associations between gastric capacity and obesity compared with populations exposed to highly processed food environments, consistent with an ecological mismatch model.

### Developmental plasticity

Early-life nutritional conditions may influence adult gastric accommodation capacity through developmental programming. Individuals exposed to early-life undernutrition followed by later caloric abundance may exhibit both increased gastric tolerance for large meals and elevated metabolic risk.

### Mechanistic pathways

Variation in gastric accommodation should correlate with differences in vagal mechanosensitivity, gut–brain signaling, and satiety hormone responses (e.g. GLP-1, PYY), providing a physiological mechanism linking gastric volume to energy intake regulation.

### Intervention response heterogeneity

Individuals with greater baseline gastric accommodation may derive disproportionate metabolic benefit from interventions that reduce gastric volume or increase gastric distension signaling (e.g. high-fiber diets, pharmacologic satiety agents, or bariatric procedures).

These predictions provide a framework for integrating evolutionary theory with clinical and physiological research and allow the present hypothesis to be evaluated through prospective, mechanistic, and cross-population studies.

## CONCLUSIONS

Human gastric physiology reflects adaptation to ecological conditions characterized by intermittent food availability, variable dietary quality, and high levels of physical activity. In both pre-agrarian and agrarian environments, greater gastric accommodation would have been advantageous for facilitating bulk intake of low-calorie foods, enabling survival during intermittent scarcity, and supporting heavy labor demands. Modern environments have inverted the selective landscape. Abundant, energy-dense foods and sedentary lifestyles transform once-adaptive traits into metabolic liabilities. In modern environments, greater gastric accommodation may facilitate overconsumption, delayed satiety, and dysregulated energy balance.

Bariatric surgery demonstrates this mismatch clearly: reducing gastric volume produces immediate metabolic improvements, underscoring the central role of gastric anatomy in appetite regulation and metabolic homeostasis. Recognizing stomach capacity as part of evolutionary mismatch reframes obesity not as a failure of willpower, but as the predictable result of ancient physiology encountering novel environments. Integrating evolutionary insight with contemporary medicine can inform more effective prevention strategies, public health policies, and therapeutic approaches to address the global metabolic crisis. Finally, as stated in the introduction, this paper should be interpreted as a hypothesis-generating narrative review. References to bariatric surgery are made solely to illustrate physiological principles and evolutionary mismatch; they do not constitute an endorsement or promotion of surgical treatment.

## Data Availability

No new data were generated or analyzed in this study. Data sharing is not applicable to this article.

## Abbreviations

BMI, Body Mass Index; DEE, Daily Energy Expenditure; GLP-1, Glucagon-Like Peptide-1; METs, Metabolic Equivalent of Task; NAFLD, Non-Alcoholic Fatty Liver Disease; NASH, Non-Alcoholic Steatohepatitis; PPIs, Proton Pump Inhibitors; PYY, Peptide YY; SCFAs, Short-Chain Fatty Acids; SSBs, Sugar-Sweetened Beverages; T2DM, Type 2 Diabetes Mellitus; USO/USOs, Underground Storage Organ(s); UPF, (Ultra-Processed Food(s); WHO, World Health Organization.
